# First Identification of Emerging Pathogenic Yeast in *Clogmia albipunctata* (Diptera: Psychodidae) at a Brazilian Hospital

**DOI:** 10.3390/microorganisms12122520

**Published:** 2024-12-07

**Authors:** Kéren Vieira-Alcântara, Thiago Pavoni Gomes Chagas, Gisela Lara da Costa, Tatiane Nobre Pinto, Manoel Marques Evangelista Oliveira, Viviane Zahner

**Affiliations:** 1 Laboratório de Simulídeos e Oncocercose & Entomologia Médica e Forense, Oswaldo Cruz Instituto, Fiocruz, Avenida Brasil 4365, Manguinhos, Rio de Janeiro 21040-360, Brazil; kerenvieira@id.uff.br; 2 Departamento de Patologia, Faculdade de Medicina, Universidade Federal Fluminense, Niterói 24220-008, Brazil; tpgchagas@id.uff.br; 3 Laboratório de Epidemiologia Molecular e Biotecnologia (LEMB), Faculdade de Farmácia, Universidade Federal Fluminense, Niterói 24241-000, Brazil; 4 Laboratory of Taxonomy, Biochemistry and Bioprospecting of Fungi, Oswaldo Cruz Institute, Fiocruz, Rio de Janeiro 21040-360, Brazil

**Keywords:** mechanical vectors, hospital associated infections, one health, drain fly, fungi

## Abstract

Psychodinae (Diptera: Psychodidae), commonly known as “drain flies”, are a subfamily of insects adapted to environments modified by humans. While often regarded as harmless, it has been reported that they may carry pathogenic microorganisms, contributing to hospital environmental contamination and potentially playing a role in healthcare-associated infections. This study aimed to investigate drain flies in a hospital setting to assess their role in carrying microbial pathogens. Twenty-six drain flies were collected from a tertiary hospital using sterile tubes and processed within two hours. The insects were identified as *Clogmia albipunctata* (Williston, 1893). Whole-body macerates were cultured on EMB media, and fungal isolates were identified using MALDI-TOF MS and ITS region sequencing. The emergent pathogen *Trichosporon asahii* was isolated, highlighting the potential role of *C. albipunctata* as a mechanical vector of fungal pathogens associated with HAIs. These findings underscore the importance of monitoring drain flies as part of infection prevention and control strategies in healthcare settings.

## 1. Introduction

*Clogmia albipunctata* (Williston, 1893), known as the “drain fly”, is a synanthropic insect that can occur in large numbers in sewage treatment plants, hospitals, bathrooms and kitchens and is therefore considered a pest [1,2]. Although this insect is not considered relevant either to public health or to the economy, it has been reported to carry bacteria and fungi of clinical importance in healthcare environments [1,3,4]. However, research investigating its role in transmitting fungal pathogens in hospitals remains limited.

Drain flies thrive in humid environments, and larvae develop in moist organic matter, where they feed on different biofilms and microorganisms. As adults, they typically fly short distances, but it has been reported that they can travel from their larval habitats and be dispersed by winds of up to 1.5 km [5,6]. Cases of accidental myiasis have been documented [7], and although the subfamily Psychodinae (Diptera: Psychodidae) is non-biting, it has been proposed that they might act in pathogen transmission within healthcare settings via contact, secretion, or excretion [8], especially among immunosuppressed patients.

HAIs (health-related infections) are one of the main causes of morbidity and mortality worldwide [9], often linked to surface contamination by bacteria, fungi, and viruses [10,11]. Over the past three decades, the incidence of fungal infections, particularly those caused by opportunistic pathogens, has risen among patients with cancer, severe burns, organ transplants, and those undergoing treatment with immunosuppressive agents. The primary risk factors for *Trichosporon asahii* infection include the use of broad-spectrum antibiotics, invasive medical devices, neutropenia, and intensive care unit (ICU) hospitalization [12]. In this context, Diptera are important carriers of microbes [13]. Proper sanitation and control, along with monitoring pathogens carried by flies, should be considered as integrated strategies for preventing and infections [14]. 

In addition, global warming has impacted ecosystems, increasingly affecting insect populations, broadening their abundance and the transmission of diseases by the microorganisms they carry. It is important to understand the interactions between animals, pathogens, humans, and the environment through a One Health approach, to better understand and address the transmission of diseases. In this context, this study aimed to monitor the presence of fungi and bacteria in the hospital environment to address the gap in knowledge regarding the role of *C. albipunctata* in hospital contamination.

## 2. Materials and Methods

### 2.1. Study Area and Insect Collection

The study was carried out at a teaching hospital in the city of Niterói, in Rio de Janeiro, Brazil. The tertiary hospital is highly complex and serves several adjacent municipalities. The insects were manually collected from different areas of the hospital using sterile tubes and transported to the laboratory within two hours. A total of 26 drain flies were collected from 5 different sites (Table 1). The insects were identified based on the observation of morphological characteristics using a stereomicroscope under aseptic conditions [15]. Three of them were reserved for further taxonomy studies. 

### 2.2. Sample Preparation

The remaining 23 drain flies were placed into microtubes containing 1 mL of 0.9% saline solution, then macerated with pestles and vortexed. The suspensions were serially diluted to a dilution factor of 10^−4^, and 100 µL of each dilution was plated onto Eosin Methylene Blue (EMB – Kasvi, PR, Brazil) agar to characterize the Gram-negative microbiota of the insects. The plates were then incubated at 35 °C for 24 h. 

### 2.3. Fungal Isolation

**Figure 1:**
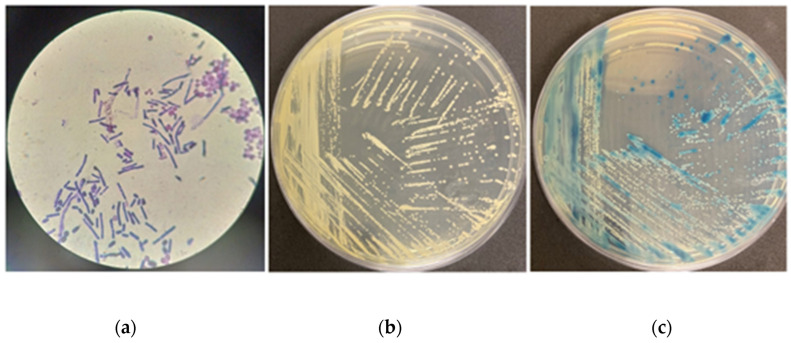
Morphological characteristics of IOCYVZM60. (**a**) Microscopic view of yeast cells after Gram stain showing pseudohyphae, arthroconidia and blastoconidia of *T. asahii* in ×1000 magnification; (**b**) Growth on Sabouraud Dextrose Agar (SDA); (**c**) Growth on CHROMagar *Candida* (BD Difco) plate. Small light blue-gray colonies were observed in the cultures suggesting non-*Candida* members.

After the incubation period, the colony-forming units were counted. A colony with morphological features suggestive of a fungal species was observed on one of the agar plates. Gram staining was performed, and microscopic observation revealed yeast-like cells in one sample (Figure 1a).

### 2.4. Fungal Culture and Identification

The sample, named IOCYVZM60, was plated onto Sabouraud Dextrose Agar (SDA) and incubated at 30 °C for 48 h, after which the morphological characteristics were reevaluated. This sample with growth on SDA was subcultured onto CHROMagar Candida (BD Difco, Franklin Lakes, NJ, USA), and colonies were analyzed following the manufacturer’s guidelines. Along with morphological testing, the isolate was further identified using molecular techniques, such as matrix-assisted laser desorption ionization–time of flight mass spectrometry (MALDI-TOF MS, Bruker™, Coventry, UK) and internal transcribed spacer (ITS) sequencing, both carried out as previously described [16]. Sequences obtained were edited with CodonCode Aligner (Genes Code Corporation, Ann Arbor, MI, USA), followed by a phylogenetic analysis using the BLAST search algorithm (https://blast.ncbi.nlm.nih.gov/Blast.cgi, accessed on 11 May 2024) to align the sequences with those in the NCBI/GenBank database.

## 3. Results

In our study, 75 Gram-negative bacteria and one sample of fungus (Table 1) were isolated from 11 of the 23 flies (Table 1). The fungal isolate was obtained from a fly captured in the exit corridor of the dining hall used by students and staff.

Colony-forming units (CFUs) on plates with microbial growth ranged from 0.1 × 10^1^ to 1.57 × 10^2^ CFU/mL. The fungal isolate, designated IOCYVZM60, exhibited growth consistent with *Trichosporon* spp. on Sabouraud Dextrose Agar (SDA) (Figure 1b). It is important to note that in the culture plate where sample IOCYVZM60 was isolated, no bacterial growth was observed. 

On CHROMagar Candida (Figure 1c) and CHROMagar Candida Plus, IOCYVZM60 developed as small, light blue-gray colonies, distinct from *Candida* species. Morphological characteristics were examined using conventional microscopy methods [17]. Analysis of both morphological and phenotypic tests confirmed characteristics consistent with the genus *Trichosporon*.

**Figure 2:**
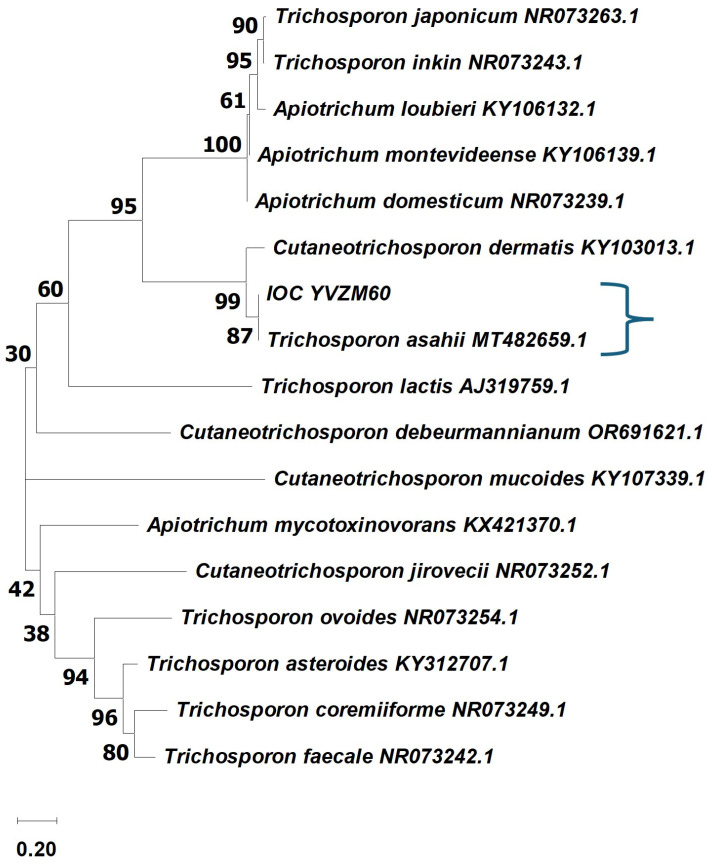
Phylogenetic analysis based on the amplification of the ITS1-5.8S-ITS2 region of ribosomal DNA using the BLAST search algorithm for comparison with the sequences deposited in the NCBI/GenBank database. The blue curly bracket shows the relationship between IOCYVZM60 and *T. asahii* MT482659.1. The numbers in the figures indicate a percentage of similarity.

The isolate was identified as *Trichosporon asahii* based on a MALDI-TOF MS score of 2.01 and ITS sequencing. ITS sequence analysis revealed 100% identity with the reference sequence of *T. asahii* (accession number PP862449.1) in the NCBI/GenBank database (Figure 2).

## 4. Discussion

This study reports, for the first time, the identification of *Trichosporon asahii* in *Clogmia albipunctata* collected from a hospital environment. The findings highlight the potential role of *C. albipunctata* in harboring and possibly disseminating fungi of clinical significance in healthcare settings. This emerging fungal pathogen was identified through MALDI-TOF MS, with results consistent with identification by ITS sequencing analysis. Herein, the MALDI-TOF MS proved to be a fast and reliable tool for identifying yeast species, and the results were in accordance with phenotypic tests.

Isolation of filamentous fungi and yeast on EMB agar has been reported previously, and growth on EMB has been shown to be greater than on sheep blood agar for pathogenic yeast *Candida glabrata* [18,19]. 

The literature is abundant with reports on the presence of bacteria, notably antibiotic-resistant strains, in non-biting insects [20,21]. However, it is scarce when it comes to fungi, especially in less-studied flies.

*C. albipunctata* is usually restricted to areas of high humidity, conditions observed where the specimens were collected. Once established, infestations are hard to eradicate, often requiring a combination of strategies to manage them effectively [22,23]. Microorganisms are able to survive in biofilms within plumbing systems and may be aerosolized, potentially exposing individuals using sinks to pathogens [24]. Other insects may feed on drain flies, and their presence could consequently contribute to the population growth of these species [25]. Studies on hospital outbreaks suggest that there could be an unexplored link between the environment of healthcare facilities and patient infections [26], underscoring the significance of findings in this study.

Species of the *Trichosporon* genus are capable of colonizing and proliferating in various regions of the human body and have been isolated from *Musca domestica* in hospital environments [27,28]. The presence of moth flies may contribute to elevated levels of indoor air contamination. In addition to enhancing allergen levels, the reproduction of these insects on biofilms can facilitate the dissemination of fungi and mycotoxins, which are byproducts of their metabolic processes [29]. Omran et al. (2020) identified various filamentous fungi in *C. albipunctata*, including *Aspergillus* spp., *Cladosporium* spp., *Penicillium* spp, *Fusarium* spp., *Mucor* spp., *Nigrospora* spp., and *Candida* spp., across different hospitals in Iran, highlighting the potential significance of these insects as reservoirs and vectors for fungal transmission [4].

Among non-Candida yeast infections, *Trichosporon* spp. are particularly significant, especially in hematological cancer patients [28]. In the ICU of a Brazilian hospital, *T. asahii* was identified as the most prevalent *Trichosporon* species isolated from urine and catheters [30]. This genus has been frequently detected in hospital environments, with a strong association between trichosporonosis and invasive clinical procedures [31]. Notably, *Trichosporon asahii* fungemia has also been reported in patients with COVID-19 [32]. The detection of *T. asahii* in *C. albipunctata*, as reported in this study, raises concerns given the unclear origins of *Trichosporon* infections. 

To minimize risks, it is advisable that hospital protocols include mitigation or regular cleaning of humid areas. These areas should have appropriate infrastructure, such as light-colored tiles, to reduce vector proliferation and facilitate hygiene practices. Continuous preventive measures are important to barrel contamination, and targeted interventions should be implemented when needed. The development of innovative pest control strategies, including biological control methods, should be prioritized. Chemical controls, while commonly used, pose environmental risks as pollutants and irritants and may be less effective against Psychodinae given their resistance to diluted surfactants [33].

A more comprehensive understanding of the influence of less-studied dipteran species on the passive transport of microorganisms could improve strategies for sanitary control. Furthermore, apart from being important decomposers in ecosystems, these insects have been recognized as sentinel organisms and can serve as an indicator of environmental contamination. Further investigation is needed to better understand the contribution of drain flies to nosocomial infections, and future research could incorporate in vivo experiments to explore the mechanisms and dynamics of microbial dissemination facilitated by these insects.

## 5. Conclusions

Despite the limitations of this study, we can conclude that drain flies can carry emerging pathogens, acting as potential reservoirs and mechanical vectors, demonstrating the need to include them in programs of surveillance and control. By monitoring fly populations, hospitals could identify potential contamination hotspots more efficiently and intervene before outbreaks occur. This perspective is expected to aid in the development of new effective strategies for vector management and the investigation of other possible sources of contamination in hospitals. Further research into the ecological roles and pathogen-carrying capacity of dipteran species is essential for improving hospital sanitation and patient safety.

## Figures and Tables

**Table 1:** Colonization of Psychodinae captured in different sites of a Brazilian hospital.

Capture Site	Moth Flies (n)	Flies With Positive Cultures	Bacterial Isolates (n)	Fungi Isolates (n)
Student and staff dining hall	8	4	47	1
Exit of outpatient blood collection	10	5	21	-
Staircase in the Faculty of Medicine	3	-	-	-
Corridor 1 of the main building	3	1	3	-
Employee time clock area	2	1	4	-

## Data Availability

The original contributions presented in this study are included in the article. Further inquiries can be directed to the corresponding author.
